# The Effect of Glucocorticoid and Mineralocorticoid Receptor Antagonists in the Skin of Aged Female Mice

**DOI:** 10.3390/ijms26178346

**Published:** 2025-08-28

**Authors:** Ameena Ali, Natalia Fossas De Mello, Yonghong Luo, Husam Bensreti, Samuel Melynk, Joseph C. Shaver, Vivek Choudhary, Meghan E. McGee-Lawrence, Wendy B. Bollag

**Affiliations:** 1 Department of Physiology, Medical College of Georgia at Augusta University, Augusta, GA 30912, USA; ameena.ali@live.mercer.edu (A.A.); 123nfossas@uccaribe.edu (N.F.D.M.); yonghongluo@hotmail.com (Y.L.); smelnyk@augusta.edu (S.M.); vivekchoudharyxyz@gmail.com (V.C.); 2 Department of Cellular Biology and Anatomy, Medical College of Georgia at Augusta University, Augusta, GA 30912, USA; hbensreti@augusta.edu (H.B.); jshaver1231@gmail.com (J.C.S.); mmcgeelawrence@augusta.edu (M.E.M.-L.); 3 VA Augusta Health Care System, Augusta, GA 30904, USA

**Keywords:** aging, aldosterone, epidermis, glucocorticoid

## Abstract

The glucocorticoid receptor (GR) and mineralocorticoid receptor (MR) are ligand-activated transcription factors that regulate epidermal homeostasis, inflammation, and function. Prior studies using epidermal-specific conditional single and double knockout mice have shown their importance in skin physiology; however, clinically human disease is largely treated pharmacologically. Our objective was to examine how systemic MR/GR antagonism affects cutaneous gene expression and epidermal thickness in aged (18-month-old) C57BL/6J female mice. Mice were treated with selective GR (relacorilant), selective MR (eplerenone), or dual GR/MR (miricorilant) antagonists for 8 weeks. Quantitative RT-qPCR analysis of the skin showed that miricorilant significantly upregulated Sgk1, a GR/MR target. Miricorilant also increased the expression of keratinocyte differentiation markers and downregulated key inflammatory cytokines and Col3a1, a collagen subtype associated with tissue remodeling. Relacorilant suppressed Scnn1g, a subunit of the epithelial sodium channel. None of the antagonists significantly altered proliferation markers, epidermal thickness, or regulators of glucocorticoid activity. Our findings show that miricorilant downregulated inflammatory cytokines and increased differentiation marker expression without affecting epidermal thickness, suggesting its potential to treat inflammatory skin diseases. The results contrast with data from GR/MR knockout studies, highlighting the likely significance of receptor dynamics. Further studies of antagonist effects on receptor interactions with co-regulators appear warranted.

## 1. Introduction

Human skin serves as a protective barrier against injury and pathogens, regulates temperature to maintain homeostasis, and supports immune defense [1]. It consists of three distinct, functional layers: epidermis, dermis, and hypodermis. The epidermis, the outermost layer, is further divided into layers from most superficial to deepest: stratum corneum, stratum granulosum, stratum spinosum, and stratum basale. Keratinocytes, the most abundant cells of the epidermis, maintain the skin barrier through proliferation and differentiation. Basal keratinocytes express proliferative markers such as keratin-5 (Krt5) and cyclin D1 (Ccnd1). As they migrate toward the skin surface, they undergo differentiation, sequentially expressing early markers like keratin-1 (Krt1) and keratin-10 (Krt10) in the suprabasal layer, followed by later differentiation markers such as involucrin (Ivl) in the upper spinous layer and loricrin (Lor) in the granular layer [2]. Beneath the epidermis is the dermis, which supports structural integrity and thermoregulation and contains appendages such as sweat glands, hair follicles, and vasculatures. The deepest layer, the hypodermis is rich in adipose tissue and serves as insulation and for structural anchoring [3].

Glucocorticoid and mineralocorticoid receptors (GR and MR, respectively) are ligand-activated transcription factors widely expressed in the skin [4]. GR suppresses inflammation by inhibiting nuclear factor kappa-light-chain-enhancer of activated B cells (NF-κB) and activator protein-1 (AP-1) signaling pathways in immune-responsive tissues, such as the skin, gut, and lung epithelium [5,6]. GR also inhibits keratinocyte proliferation, contributing to its clinical efficacy in treating inflammatory dermatoses. However, chronic GR activation can lead to skin atrophy and delayed healing [7]. MR, traditionally associated with electrolyte regulation, is also present in non-classical tissues such as the skin [8].

Laboratory studies support a role for MR in cutaneous homeostasis. In UV-exposed mice with metabolic syndrome, MR and its downstream effector gene, Sgk1, are elevated alongside oxidative stress and inflammation, which are both attenuated by spironolactone [9]. Furthermore, in human skin, aldosterone increases collagen and elastin production, while MR antagonists reduce collagen and enhance elastin [10]. These findings suggest that MR antagonists might improve skin elasticity. Notably, glucocorticoids have a higher affinity for MR than GR, but their activity is regulated by the enzyme 11-beta-hydroxy-steroid dehydrogenase type 2 (11β-HSD2), which inactivates cortisol in humans. 11β-HSD2 and 11β-hydroxysteroid dehydrogenase type 1 (11β-HSD1), which activates inactive cortisone, are both essential for regulating receptor activity [11].

Knockout mouse models reveal distinct and overlapping roles for GR and MR. GR knockout, specifically in keratinocytes, leads to defective epidermal barrier formation with thinner skin, altered differentiation, and increased inflammatory signaling, resembling atopic dermatitis and psoriasis [12,13]. Epidermal-specific MR knockout mice have increased keratinocyte proliferation and differentiation with resistance to glucocorticoid-induced atrophy, although ablation of MR increases cytokine levels and NF-κB activity, implicating MR in anti-inflammatory regulation [14]. MR also modulates GR genomic binding in keratinocytes, with MR knockout reducing GR DNA binding by 35% [15].

As glucocorticoids activate MR, glucocorticoids’ adverse effects, such as skin atrophy, may be MR mediated [14]. MR antagonists can improve wound healing and limit steroid-induced skin atrophy in mice and in human skin explants [16]. Moreover, topical co-application of the MR antagonist spironolactone with the glucocorticoid clobetasol significantly limits clobetasol-induced atrophy [17]. Dual GR/MR knockout mice display severe epidermal differentiation defects at birth, as well as psoriasis-like inflammation, suggesting that GR and MR both coordinate epidermal homeostasis [5].

Prior GR/MR knockout mice studies have shown their importance in skin physiology; however, the effects of pharmacological inhibition remain unclear. The goal of this study was to investigate the effects of MR and GR antagonists on keratinocyte differentiation, proliferation, inflammation, and skin barrier function in mouse skin. As genetic knockout of MR and GR is not feasible in humans, pharmacologic antagonist models offer a more translatable approach to human skin physiology. Therefore, to test our hypothesis, we administered vehicle or a GR antagonist, relacorilant; MR antagonist, eplerenone; or a dual GR/MR antagonist, miricorilant, to 18-month-old female mice for 8 weeks and measured various parameters in their skin.

## 2. Results

### 2.1. The Effects of MR and GR Antagonists on mRNA Expression of MR/GR Markers in Skin Isolated from Vehicle or RELA-, EPL-, or MIRI-Treated Mice

**Figure 1:**
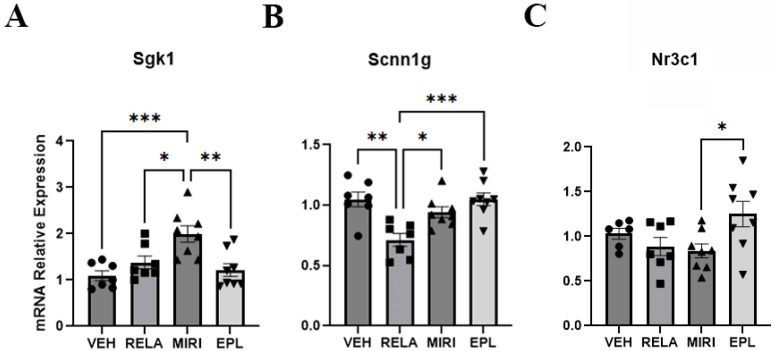
The effects of RELA, MIRI, and EPL on the mRNA expression of Sgk1, Scnn1g, and Nr3c1. (**A**) Eighteen-month-old female C57BL/6 mice were randomly assigned to receive chow with vehicle (VEH; Teklad 2018) or the glucocorticoid receptor (GR) antagonist relacorilant (RELA; 60 mg/kg/day), the mineralocorticoid receptor (MR) antagonist eplerenone (EPL; 200 mg/kg/day), or the dual GR/MR antagonist miricorilant (MIRI; 60 mg/kg/day) for 8 weeks. After mouse sacrifice, dorsal skin was collected for RNA isolation and analyzed by RT-qPCR. Sgk1 mRNA expression was significantly increased by MIRI treatment compared with all treatments. (**B**) Scnn1g mRNA expression was significantly decreased by RELA treatment compared with all treatments. (**C**) Nr3c1 mRNA expression was significantly increased by EPL compared only to MIRI treatment. The data were analyzed with the delta-delta Ct method (2^−ΔΔCt^) as described in Section 4; n = 7–8; * *p* ≤ 0.05, ** *p* ≤ 0.01 and *** *p* ≤ 0.001 as indicated.

We first tested whether relacorilant (RELA; selective GR antagonist), eplerenone (EPL; selective MR antagonist) or miricorilant (MIRI; selective dual GR and MR antagonist) influenced mRNA expression of GR/MR markers. Specifically, we measured the expression of serum/glucocorticoid-regulated kinase 1 (Sgk1), sodium channel epithelial 1 subunit alpha and gamma (Scnn1a, Scnn1g), and nuclear receptor subfamily 3, group C, member 1 and member 2 (Nr3c1 and Nr3c2 encoding GR and MR, respectively). Based on the RT-qPCR results, we found that MIRI significantly increased the mRNA expression of Sgk1, a downstream marker of GR/MR activity, compared with VEH and other treatment groups. The selective MR antagonist (EPL) and selective GR antagonist (RELA) had no significant effect on the expression of Sgk1, which points to the idea that both GR and MR may need to be inhibited simultaneously to observe this outcome, suggesting overlapping functions of these two receptors in the skin (Figure 1A).

As shown in Figure 1B, RELA significantly decreased the expression of Scnn1g, a subunit of the epithelial sodium channel (ENaC). MIRI- and EPL-treated skin displayed Scnn1g expression levels comparable to VEH (Figure 1B). The expression levels of Scnn1a, another subunit of ENaC, were not significantly altered by MIRI, RELA, or EPL (Supplementary Figure S1). On the other hand, EPL treatment significantly increased the expression of Nr3c1 (encoding GR) when compared with MIRI (Figure 1C). This result suggests a possible compensatory upregulation of GR when MR is inhibited compared with dual GR/MR inhibition, which leads to a decreased expression of Nr3c1 (that did not achieve significance relative to expression upon vehicle treatment). MIRI, RELA, and EPL did not significantly change the expression of Nr3c2 (encoding MR; Supplementary Figure S1).

### 2.2. The Effects of MR and GR Antagonists on mRNA Expression of Keratinocyte Proliferation and Differentiation Markers and Epidermal Thickness in Skin Isolated from Vehicle- or RELA-, EPL, or MIRI-Treated Mice

**Figure 2:**
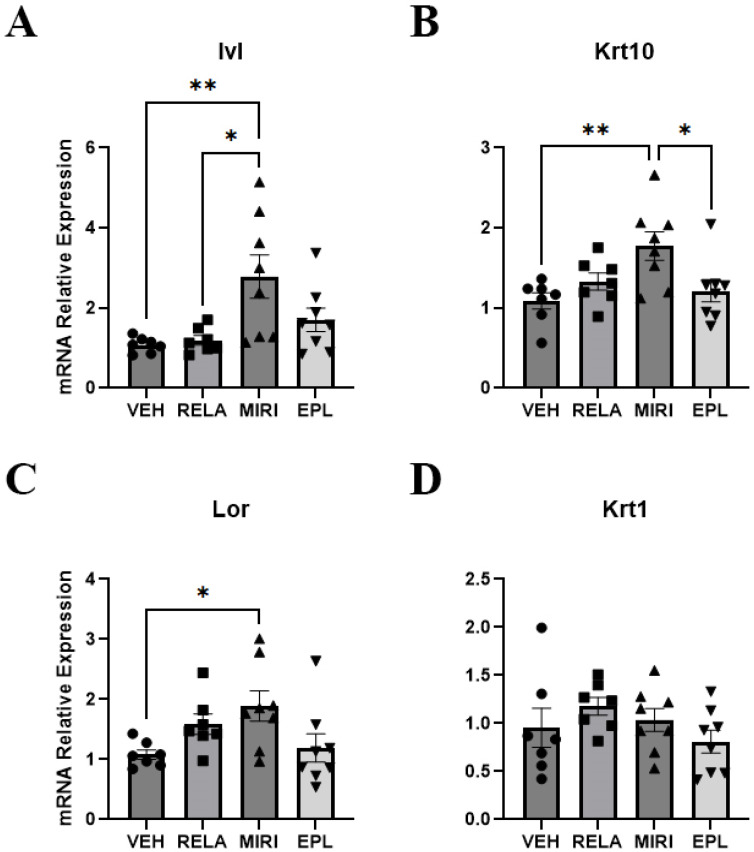
The effect of RELA, MIRI, and EPL on mRNA expression of keratinocyte differentiation markers. Mice were treated, skin samples collected and RNA isolated for RT-qPCR as in Figure 1. (**A**) Ivl expression was significantly upregulated in MIRI-treated mice compared with those receiving VEH or RELA. (**B**) Krt10 expression was significantly upregulated in MIRI-treated mice compared with treatment with VEH and EPL. (**C**) Lor expression was significantly upregulated in MIRI-treated mice compared with VEH control. (**D**) Krt1 mRNA expression was not significantly altered by RELA, MIRI or EPL. n = 7–8; * *p* ≤ 0.05 and ** *p* ≤ 0.01 as indicated.

**Figure 3:**
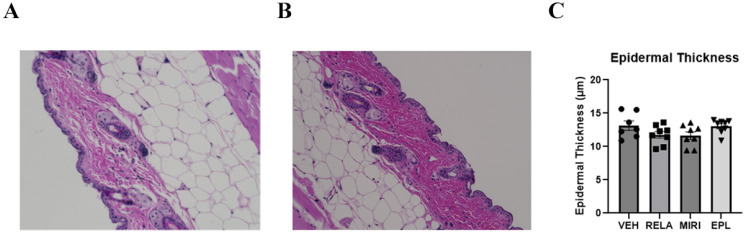
The effect of RELA, MIRI, and EPL on epidermal thickness. There was no significant difference measured with any treatment. (**A**,**B**) Representative images of epidermal thickness in skin sections stained with H&E. (**C**) Epidermal thickness was quantified by blinded observers in sections from each mouse as described in Section 4. Average epidermal thickness values from each mouse were analyzed by ANOVA and Tukey post-hoc tests. Results are shown as means ± SEM in micrometers (μm); n = 7–8.

Markers of keratinocyte proliferation that were monitored included aquaporin-3 (Aqp3) and keratin-5 (Krt5), which are expressed predominantly in proliferating basal keratinocytes, and cyclin D1 (Ccnd1), a regulator of the cell cycle increased in proliferating cells [18,19]. RELA, EPL, and MIRI did not significantly alter the expression of any of these keratinocyte proliferation markers (Supplementary Figure S1). The markers of keratinocyte differentiation examined included involucrin (Ivl), keratin-1 (Krt1), keratin-10 (Krt10), and loricrin (Lor). MIRI significantly increased the mRNA expression of Ivl, Krt10, and Lor (Figure 2). GR inhibition (RELA) or MR inhibition (EPL) alone had no significant effect on any of these differentiation markers; however, blocking both GR and MR together seemed to promote differentiation uniquely, suggesting that the combined activation of GR and MR may stimulate proliferation and/or inhibit differentiation. Furthermore, skin epidermal thickness is sustained through basal keratinocyte proliferation, migration, and differentiation to the other layers of the epidermis. Therefore, we assessed for skin epidermal thickness by H&E staining of sections of harvested dorsal skin. Nevertheless, there was not a significant change in epidermal thickness with any of the antagonists (Figure 3).

### 2.3. The Effects of MR and GR Antagonists on mRNA Expression of Inflammation Markers in the Skin Isolated from Vehicle-, RELA-, EPL-, or MIRI-Treated Mice

**Figure 4:**
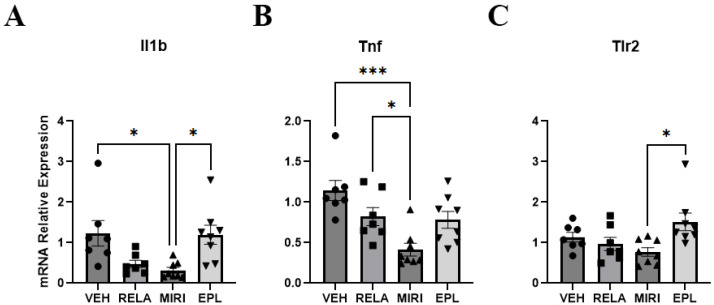
The effect of RELA, MIRI, and EPL on cutaneous mRNA expression of inflammatory mediators. Inflammatory mediator mRNA expression was monitored in skin samples from mice treated as in Figure 1. (**A**) Il1b expression was significantly downregulated in MIRI-treated mice compared with those treated with VEH or EPL. (**B**) Tnf expression was significantly downregulated in MIRI-treated mice relative to VEH- and RELA-administered mice. (**C**) Tlr2 expression was significantly upregulated in EPL-treated mice compared with MIRI-treated animals. n = 7–8; * *p* ≤ 0.05 and *** *p* ≤ 0.001 as indicated.

Glucocorticoid-activated GR is known to exert anti-inflammatory effects in multiple organs of the body, whereas MR promotes inflammation in some tissues [20,21]. Therefore, we also measured the cutaneous expression of markers of inflammation including interleukin-1α (Il1a), interleukin-1β (Il1b), interleukin-6 (Il6), and tumor necrosis factor (Tnf), as well as toll-like receptor 4 (Tlr4) and toll-like receptor 2 (Tlr2), pattern recognition receptors that can stimulate the innate immune system. MIRI significantly decreased the expression of Il1b and Tnf (Figure 4A,B). These results indicate that dual GR/MR inhibition may reduce inflammation in the skin. EPL also significantly increased the expression of Tlr2 compared with MIRI (Figure 4C). As MR antagonism increased Tlr2 expression, MR may suppress Tlr2 expression under normal conditions. There was no significant change in Il1a, Il6, or Tlr4 expression (Supplementary Figure S2).

### 2.4. The Effects of MR and GR Antagonists on mRNA Expression of MR/GR Activity Regulators in Skin Isolated from Vehicle- or RELA-, EPL-, or MIRI-Treated Mice

**Figure 5:**
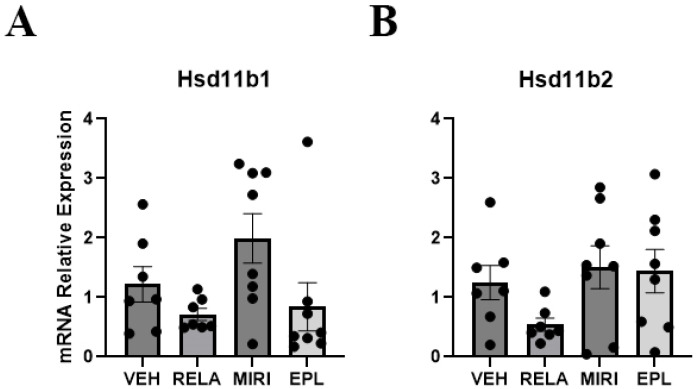
The effect of RELA, MIRI, and EPL on mRNA expression of MR/GR activity regulators. The mRNA levels of glucocorticoid-activating and -inactivating enzymes were monitored in skin from mice treated as in Figure 1. (**A**) Hsd11b1 mRNA expression was not significantly altered by RELA, MIRI, or EPL treatment. (**B**) Hsd11b2 mRNA expression was not significantly altered by RELA, MIRI, or EPL treatment. n = 7–8.

Key regulators of MR/GR activity are 11β-hydroxysteroid dehydrogenase type 1 (encoded by Hsd11b1) and 11β-hydroxysteroid dehydrogenase type 2 (encoded by Hsd11b2). In mice, Hsd11b1 converts inactive 11-dehydrocorticosterone to active corticosterone, whereas Hsd11b2 catalyzes inactivation of corticosterone by its metabolism to 11-dehydrocorticosterone. Our results show that the cutaneous expression of Hsd11b1 and Hsd11b2 was not significantly altered by any treatment (Figure 5), suggesting that GR and MR do not regulate the transcription of these glucocorticoid activity-regulating enzymes in the skin under normal conditions.

### 2.5. The Effects of MR and GR Antagonists on mRNA Expression of Dermal Extracellular Matrix Markers in Skin Isolated from Vehicle-, RELA-, EPL-, or MIRI-Treated Mice

**Figure 6:**
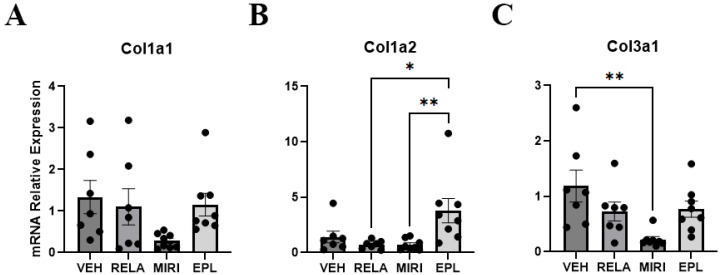
The effect of RELA, MIRI, and EPL on mRNA expression of dermal collagens. The expression of dermal extracellular matrix collagens in skin samples obtained from mice treated as in Figure 1. (**A**) None of the treatments significantly changed the expression of Col1a1. (**B**) EPL significantly increased the expression of Col1a2 compared with MIRI and RELA. The change in expression did not achieve significance with respect to VEH. (**C**) MIRI significantly decreased the expression of Col3a1compared with VEH. n = 7–8; * *p* ≤ 0.0 and ** *p* ≤ 0.01 as indicated.

To determine if GR and MR affected the function of the dermis in addition to the epidermis, we determined the expression of dermal extracellular matrix markers collagen type 1 alpha 1 (Col1a1), collagen type 1 alpha 2 (Col1a2), and collagen type 3 alpha 1 (Col3a1). Col1a1 and Col1a2 expressed in the skin provide tensile strength, whereas Col3a1 imparts flexibility [22]. Based on our RT-qPCR results, there was no significant change in expression of Col1a1 with any treatment (Figure 6A). Regarding Col1a2, EPL significantly increased its expression compared with MIRI and RELA. However, the change in expression did not achieve significance compared with VEH, suggesting that MR’s influence on Col1a2 expression under basal conditions is not marked (Figure 6B). MIRI significantly decreased the expression of Col3a1 compared with VEH (Figure 6C). This result suggests that GR and MR together may play a role in regulating Col3a1 expression.

## 3. Discussion

In this study, we found that dual glucocorticoid and mineralocorticoid receptor antagonism with miricorilant (MIRI) had distinct effects on epidermal gene expression compared with selective GR or MR antagonism alone (summarized in Table 1). MIRI treatment significantly increased the expression of Sgk1, a marker of GR/MR signaling. This result is consistent with Sgk1 being a downstream target of both GR and MR, requiring inhibition of both to detect an effect, although it is somewhat counterintuitive to the idea that Sgk1 is an MR/GR target. However, Sgk1 is regulated by factors in addition to GR and MR, including hormones like gonadotropins and growth factors such as transforming growth factor-β, suggesting the possibility that the cell is trying to compensate for the antagonist-mediated inhibition of MR/GR activity by upregulating the levels of its effector through an alternate, unknown mechanism [23].

Unexpectedly, perhaps, MIRI showed anti-inflammatory effects in terms of decreasing the expression of inflammatory markers Il1b and Tnf. IL-1β and TNF-α are inflammatory cytokines and transcriptional targets of NF-κB [24]. GR activation has been shown to suppress inflammation through the NF-κB pathway, and deficiency of GR or MR in mice results in susceptibility for the development of inflammatory skin lesions, whereas deficiency of both receptors further exacerbates inflammation [5,8] and reviewed in [25]. Their downstream effector Sgk1 has also been reported to exert anti-inflammatory effects in the imiquimod-induced mouse model of psoriasis. Thus, Sgk1 inhibition exacerbates skin inflammation, increases the secretion of cytokines, including IL1β, IL6 and TNFα, and promotes Th17 cell infiltration into the skin; furthermore, the non-specific MR antagonist spironolactone ameliorates this phenotype [26]. Dong et al. [27] have also determined an ability of Sgk1 inhibition to worsen not only imiquimod responses but also the inflammation elicited by calcipotriol (a vitamin D analog) in an atopic dermatitis model. In addition, these authors found that Sgk1 inhibition enhances macrophage polarization to an inflammatory (M1) phenotype and that Sgk1 is downregulated in macrophages in skin samples from patients with psoriasis and atopic dermatitis [27]. These data support the idea that, in the skin, GR, MR, and Sgk1 all suppress inflammation, and that the observed upregulation of Sgk1 expression with MIRI treatment might be an attempt to compensate for the inhibition of GR and MR by MIRI.

On the other hand, GR inhibition with relacorilant (RELA) selectively decreased the expression of Scnn1g, a subunit of the epithelial sodium channel (ENAC) (Figure 1); this finding is perhaps unexpected, considering that ENAC is generally considered to be an MR target, although the fact that GR and MR bind to many of the same hormone response elements may explain the result [28]. Additionally, MIRI, but not RELA or eplerenone (EPL), enhanced the expression of keratinocyte differentiation markers such as Ivl, Krt10, and Lor, suggesting that both MR and GR may regulate the differentiation process in a redundant manner. Notably, none of the treatments significantly altered markers of proliferating keratinocytes (Aqp3, Krt5, Ccnd1) or regulators of MR/GR activity (Hsd11b1, Hsd11b2). Regarding the dermis, MIRI treatment reduced the expression of Col3a1, a dermal extracellular matrix component, without any significant effect on Col1a1 or Col2a1. It is important to note that our findings with receptor antagonism differ compared with epidermal-specific receptor knockout mouse models. One potential explanation for the disparity is the fact that the antagonists will inhibit MR and/or GR in multiple cell types in the skin and elsewhere due to their systemic delivery. However, it is also important to highlight these differences by discussing how receptor antagonism can potentially influence receptor dynamics, transcriptional regulation, and compensatory signaling leading to distinct outcomes compared with complete receptor loss, as discussed below.

Pharmacologic blockade of GR and MR with MIRI does not replicate the phenotype observed in epidermal-specific GR/MR double knockout mice (DKO), in which loss of these two genes results in thicker, more proliferative epidermis and extensive inflammation [5]. Thus, MIRI upregulated keratinocyte differentiation markers such as Ivl, Krt, and Lor, suggesting enhanced differentiation. In contrast, DKO mice displayed unorganized keratinocytes with poorly differentiated strata granulosum and corneum, consistent with impaired epidermal maturation. Keratinocyte proliferation was also differentially affected. MIRI, EPL, and RELA had no significant effect on proliferation-related genes (Aqp3, Krt5, Ccnd1), whereas MR/GR double knockout (DKO) mice exhibit increased keratinocyte proliferation, although it is possible that this effect could be related to inflammation, which often leads to hyperplasia in inflammatory skin disorders [7,29]. Additionally, Nguyen et al. (2016) have reported that MR antagonism rescued keratinocyte proliferation in mice with GC-induced skin atrophy or diabetes [16]. However, our study with healthy, aged female mice did not show such changes, suggesting that the proliferative effects of MR antagonism may be context- or disease-dependent. Furthermore, Sevilla and Perez (2018) also found that expression of Hsd11b2, a corticosteroid regulator, is reduced in epidermal-specific single knockout MR mice, indicating that Hsd11b2 expression is MR dependent [7]. In contrast, in our study, MIRI, EPL and RELA treatments did not alter Hsd11b1 or Hsd11b2 expression. Taken together, these differences highlight that receptor antagonism and genetic deletion have distinct effects on GR/MR signaling pathways. Antagonists block receptor activation but preserve receptor protein expression, allowing for potential partial signaling, compensatory feedback, or interactions with co-regulators (co-activators and/or co-repressors). In contrast, genetic knockouts eliminate the receptor entirely, potentially allowing coactivators/corepressors that normally interact with MR and/or GR to instead bind to and regulate the activity of other nuclear hormone receptors.

There are multiple reasons why antagonism might lead to distinct effects compared with receptor deletion, as a result of differences in transcriptional regulation, receptor dynamics, and/or compensatory signaling mechanisms. Both GR and MR affect transcription of their downstream genes by binding to hormone response elements in target genes and recruiting various coactivators or corepressors to modulate gene expression; thus, as noted above, ablation of these genes could potentially allow these coactivators/corepressors to bind to and regulate the activity of other nuclear hormone receptors. Our results show that MIRI significantly increased the mRNA expression of Sgk1, a marker of GR/MR activity, which suggests that the Sgk1 gene may be regulated in a complex manner and/or be upregulated through a compensatory mechanism, as mentioned previously, or via a partial agonist activity. Indeed, antagonists may not “turn off” receptor function completely, and instead, they can induce distinct conformations that displace co-activators and recruit co-repressors rather than inducing a complete loss of receptor function [30,31]. For example, a study by Hartmann et al. (2021) demonstrated how there can be crosstalk between MR and GR. MR plays an important role in maintaining baseline expression of Fkbp5, a co-chaperone that modulates GR sensitivity [32]. When MR is deleted or pharmacologically inhibited, Fkbp5 expression is reduced, leading to increased GR sensitivity and an exaggerated transcriptional response to stress-induced glucocorticoids. Similarly, Alvarez de la Rosa et al. (2024) discussed that MR and GR frequently form heteromeric complexes and share overlapping DNA response elements but possess distinct transactivation domains and ligand sensitivities [33]. These shared signaling components allow for mutual modulation, such that the loss or inhibition of one receptor can shift these signaling factors to the other, or perhaps even to another nuclear hormone receptor, such as the vitamin D receptor (VDR), which is known to regulate keratinocyte differentiation [34]. This dynamic crosstalk suggests that pharmacologic antagonism may elicit subtler or context-specific responses compared with the complete absence of a receptor, as in knockout models. On the other hand, antagonists can also exert off-target effects, which could make them either more or less beneficial for treating a particular disease, depending on whether the other signaling targets reinforce or oppose the effects of the desired target and/or result in adverse side effects.

In conclusion, it is important to further investigate GR and MR antagonism to understand the full spectrum of effects of these inhibitors on skin biology. Antagonists are already used clinically to treat various endocrine and inflammatory conditions, but their impact on skin health and homeostasis is not fully understood. Our findings show that dual GR/MR antagonism with miricorilant reduced inflammatory gene expression and promoted keratinocyte differentiation in female mice. These are two effects could be potentially beneficial in inflammatory skin diseases with impaired barrier function, such as atopic dermatitis, psoriasis, or chronic wounds. Based on these and other studies, the effects of antagonism in models of inflammation, aging, and metabolic dysfunction, where corticosteroid signaling is often dysregulated, appear warranted. However, changes in gene expression are not always translated into effects at the protein level. Future studies should investigate downstream protein expression and enzyme activities to define the biological consequences of receptor blockade. It would also be useful to explore how GR and MR regulate epidermal processes such as collagen remodeling, wound repair, and barrier healing under both healthy and diseased conditions. A deeper understanding of the molecular mechanisms underlying GR/MR crosstalk could help optimize therapeutic strategies and minimize unwanted effects in clinical settings.

## 4. Materials and Methods

### 4.1. Animal Experiments

Forty 18-month-old C57BL/6 female mice were obtained from the NIA rodent colony (National Institute on Aging, Bethesda, MD, USA); one mouse died prior to the onset of the study due to unknown causes. Aged mice were used because glucocorticoids, GR, and MR are thought to play a role in skin aging [26]. Mice were group-housed in an accredited facility and maintained on a 12 h light/dark cycle at a temperature between 20 and 25 °C with ad libitum access to food and water. All procedures were conducted in accordance with NIH guidelines and approved by the Augusta University Institutional Animal Care and Use Committee (IACUC) protocol number #2014-0673 (with an approval date of 27 July 2023). Mice were allowed to acclimate for one week prior to the start of the experiment. Animals were then randomly assigned to receive one of four diets for eight weeks: (1) control base diet (VEH; Teklad 2018); (2) chow containing the selective mineralocorticoid receptor (MR) antagonist, eplerenone (EPL; 200 mg/kg/day); (3) chow containing the selective glucocorticoid receptor (GR) antagonist, relacorilant (RELA; 60 mg/kg/day); or (4) chow containing the dual GR/MR antagonist, miricorilant (MIRI; 60 mg/kg/day). RELA and MIRI are water-insoluble, necessitating diet-based (rather than water-based or injectable) administration. The dose for EPL was selected based on published studies demonstrating effective MR antagonism at this level [35,36,37]. Doses for RELA and MIRI were determined based on manufacturer recommendations. Mice were assigned numerical identifiers to ensure blinding during downstream analyses.

Body mass and food consumption were monitored throughout the treatment period. Mice were provided food and water ad libitum under standard conditions, specified by National Institutes of Health (NIH) and Association for Assessment and Accreditation of Laboratory Animal Care (AALAC) guidelines. At the end of the study, all mice were euthanized under isoflurane anesthesia to minimize stress-related confounding. Tissues were collected immediately after sacrifice. Nine mice exhibited pathological conditions often associated with age (e.g., tumors) and thus were excluded from further analyses. The final group numbers were (VEH = 7, RELA = 7, MIRI = 8, and EPL = 8).

### 4.2. Sample Preparation

Each frozen skin sample was thawed, placed in a Petri dish, cut into smaller fragments weighing approximately 0.02 g, and transferred into an Eppendorf microtube with 0.1 g of zirconium oxide beads (2.5 mm:1.0 mm diameter, 1:1) from Next Advance (Troy, NY, USA) on ice. Cold TRIzol (ThermoFisher Scientific, Waltham, MA, USA) was then added to the tubes, and the samples were homogenized in a cold room using a Bullet Blender (Next Advance, Inc., Troy, NY, USA) at speed 6 for a total of 9 min, divided into two intervals of 6 and 3 min, until the sample pieces could no longer be observed.

### 4.3. RNA Isolation and RT-qPCR

After homogenization, chloroform was introduced into each sample, followed by centrifugation at 13,200× *g* at 4 °C for 15 min to facilitate phase separation. The upper phase was carefully extracted using a pipette in three successive transfers and collected into a fresh microtube. RNA was purified with PureLink™ RNA Mini Kits (ThermoFisher Scientific). Then, a NanoDrop 2000c spectrophotometer (ThermoFisher Scientific) was used to measure the RNA concentration and purity (A_260nm_/A_280nm_) with 1 µL of RNA. RNA was converted to cDNA using a high-capacity cDNA reverse transcription kit with RNase inhibitor (ThermoFisher Scientific). cDNA was placed in a 96-well plate with Taqman primer-probe sets (ThermoFisher Scientific) for quantitative RT-PCR using a StepOnePlus (ThermoFisher Scientific) instrument with a reaction volume of 10 µL. The data were analyzed with the delta-delta Ct method (2^−ΔΔCt^) using the average value of the two housekeeping genes Rlpl0 and Ppia. The stability of the Ct value for the housekeeping genes was also examined to verify consistency of these values among samples. The primer-probe sets used in this study are shown in Table 2.

### 4.4. Determination of Epidermal Thickness

Formalin-fixed skin samples were paraffin embedded, sectioned (5 µm) and stained with hematoxylin and eosin (H&E). Each sample included two skin sections per mouse per slide. For each stained sample of skin, at least 3 randomly selected fields (images) were photographed through an EVOS^TM^ M5000 imaging system (ThermoFisher Scientific). In each image, three perpendicular measurements of the epidermis were taken and averaged to generate one representative value per image. The three image-level values from each section were then averaged to yield a single epidermal thickness value per section. Finally, the values from the two sections were averaged to obtain a single epidermal thickness value per sample. Measurements were performed in a blinded manner by two independent investigators using ImageJ 1.54j Software (National Institutes of Health, Bethesda, MD, USA) and averaged. Final sample values were used to compare experimental groups using ANOVA with Tukey post-hoc tests using Graphpad Prism version 10.4.1 (Boston, MA, USA).

## Figures and Tables

**Table 1:** Summary of RELA, MIRI, and EPL treatment on mRNA expression of all genes tested. MIRI increased the expression of the MR and GR marker Sgk1 as well as the keratinocyte differentiative markers Ivl, Krt10, and Lor (blue). MIRI decreased the expression of inflammatory mediators Il1b and Tnf and the dermal ECM marker Col3a1 (red). RELA decreased the expression of the MR/GR target Scnn1g (fuchsia). EPL increased the expression of Nr3c1, Tlr2 and Col2a1 relative to MIRI and/or RELA (purple).

Markers of MR/GR	Proliferative KCs	Differentiative KCs	Inflammatory Markers	Regulators of MR/GR Activity	Dermal ECM
Sgk1 (↑MIRI)	Aqp3	Ivl (↑MIRI)	Il1a	Hsd11b1	Col1a1
Scnn1a	Krt5	Krt1	Il1b (↓MIRI)	Hsd11b2	Col2a1 (EPL > MIRI, RELA)
Scnn1g (↓RELA)	Ccnd1	Krt10 (↑MIRI)	Il6		Col3a1 (↓MIRI)
Nr3c1 (EPL > MIRI)		Lor (↑MIRI)	Tnf (↓MIRI)		
Nr3c2			Tlr4		
			Tlr2 (EPL > MIRI)		

**Table 2:** List of primer-probe sets used for RT-qPCR.s.

Primer Name	Source	Identifier
Sgk1	ThermoFisher Scientific	Mm00441380_m1
Scnn1a	ThermoFisher Scientific	Mm00803386_m1
Scnn1g	ThermoFisher Scientific	Mm00441228_m1
Nr3c1	ThermoFisher Scientific	Mm00433832_m1
Nr3c2	ThermoFisher Scientific	Mm01241596_m1
Aqp3	ThermoFisher Scientific	Mm01208559_m1
Krt5	ThermoFisher Scientific	Mm01305291_g1
Ccnd1	ThermoFisher Scientific	Mm00432359_m1
Ivl	ThermoFisher Scientific	Mm00515219_s1
Krt1	ThermoFisher Scientific	Mm00492992_g1
Krt10	ThermoFisher Scientific	Mm03009921_m1
Lor	ThermoFisher Scientific	Mm01219285_m1
Il1a	ThermoFisher Scientific	Mm00439620_m1
Il1b	ThermoFisher Scientific	Mm01336189_m1
Il6	ThermoFisher Scientific	Mm99999064_m1
Tnf	ThermoFisher Scientific	Mm00443259_m1
Tlr4	ThermoFisher Scientific	Mm00445273_m1
Tlr2	ThermoFisher Scientific	Mm01213946_g1
Hsd11b1	ThermoFisher Scientific	Mm00476182_m1
Hsd11b2	ThermoFisher Scientific	Mm01251104_m1
Col1a1	ThermoFisher Scientific	Mm00801666_g1
Col1a2	ThermoFisher Scientific	Mm000483888_m1
Col3a1	ThermoFisher Scientific	Mm00802300_m1

## Data Availability

Data generated during the study will be provided upon reasonable request to the corresponding author.

## Supplementary Materials

The following supporting information can be downloaded at: https://www.mdpi.com/article/10.3390/ijms26178346/s1.
