# Supplementary material for: The Effect of Glucocorticoid and Mineralocorticoid Receptor Antagonists in the Skin of Aged Female Mice
Source: Int J Mol Sci. 2025 Aug 28;26(17):8346. doi: 10.3390/ijms26178346 (PMC12427858; doi:10.3390/ijms26178346)
Supplement: Supplementary file 1 [file ijms-26-08346-s001.zip › ijms-3785742-supplementary.pdf]

<u>**Supplementary Materials**</u>

for

The Effect of Glucocorticoid and Mineralocorticoid Receptor Antagonists in the Skin of Aged Female Mice

by

Ameena Ali, Natalia Fossas de Mello, Yonghong Luo, Husam Bensreti, Samuel Melynk, Joseph C. Shaver, Vivek Choudhary, Meghan E. McGee-Lawrence and Wendy B. Bollag

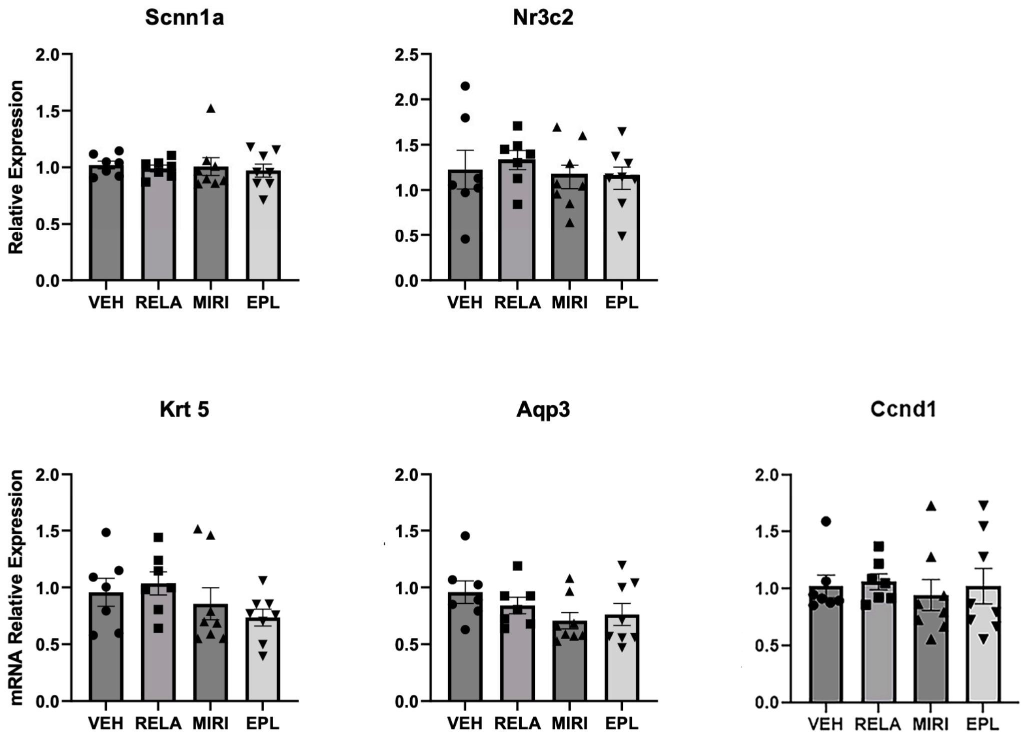

Figure S1. RELA, MIRI, and EPL had no effect on the mRNA expression of MR/GR-responsive genes, Scnn1a and Nr3c2 or markers of proliferative keratinocytes, Krt5, Aqp3 and Ccnd1. Eighteen-month-old female C57BL/6 mice were randomly assigned to receive chow with vehicle (VEH; Teklad 2018) or the glucocorticoid receptor (GR) antagonist relacorilant (RELA; 60 mg/kg/day), the mineralocorticoid receptor (MR) antagonist eplerenone (EPL; 200 mg/kg/day), or the dual GR/MR antagonist miricorilant (MIRI; 60 mg/kg/day) for 8 weeks. After mouse sacrifice, dorsal skin was collected for RNA isolation and analyzed by RT-qPCR. The data were analyzed with the delta-delta Ct method ($2^{-\Delta\Delta Ct}$) as described in the Materials and Methods section; n = 7–8; * $p \leq 0.05$, ** $p \leq 0.01$ and *** $p \leq 0.001$ as indicated.

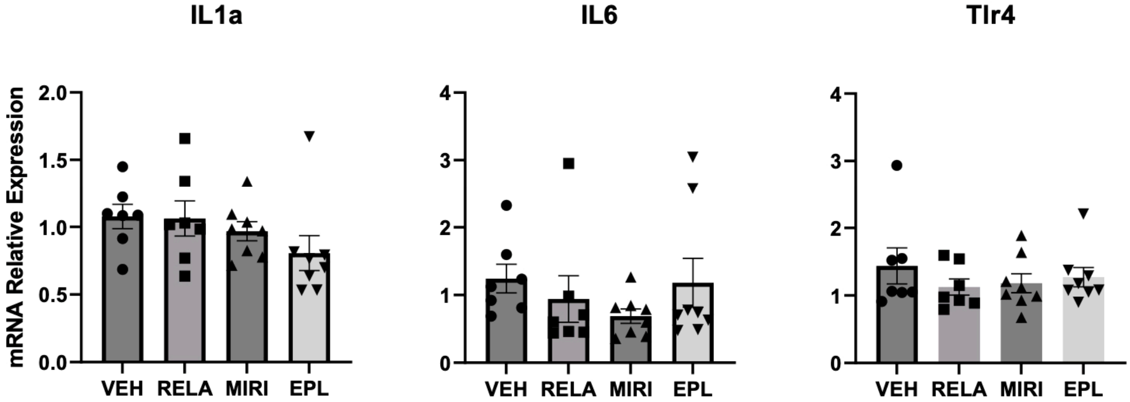

Figure S2. RELA, MIRI, and EPL had no effect on the mRNA expression of inflammatory mediators, Il1a, Il6, and Tlr4. Eighteen-month-old female C57BL/6 mice were randomly assigned to receive chow with vehicle (VEH; Teklad 2018) or the glucocorticoid receptor (GR) antagonist relacorilant (RELA; 60 mg/kg/day), the mineralocorticoid receptor (MR) antagonist eplerenone (EPL; 200 mg/kg/day), or the dual GR/MR antagonist miricorilant (MIRI; 60 mg/kg/day) for 8 weeks. After mouse sacrifice, dorsal skin was collected for RNA isolation and analyzed by RT-qPCR. The data were analyzed with the delta-delta Ct method ($2^{-\Delta\Delta Ct}$) as described in the Materials and Methods section; n = 7–8; * $p \leq 0.05$, ** $p \leq 0.01$ and *** $p \leq 0.001$ as indicated.
